# Cat rabies in Brazil: a growing One Health concern

**DOI:** 10.3389/fpubh.2023.1210203

**Published:** 2023-07-19

**Authors:** Jonathan Santos de Lima, Enio Mori, Louise Bach Kmetiuk, Leandro Meneguelli Biondo, Paulo Eduardo Brandão, Alexander Welker Biondo, Paulo César Maiorka

**Affiliations:** ^1^Department of Pathology, Faculty of Veterinary Medicine and Animal Sciences, University of São Paulo, São Paulo, Brazil; ^2^Pasteur Institute, São Paulo, SP, Brazil; ^3^Department of Veterinary Medicine, Federal University of Paraná, Curitiba, PR, Brazil; ^4^National Institute of the Atlantic Forest (INMA), Brazilian Ministry of Science, Technology, and Innovation, Santa Teresa, Espírito Santo, Brazil

**Keywords:** neglected tropical diseases, rabies control, rabies surveillance, spillover, zoonosis

## Abstract

This review of human and cat rabies from 1986 to 2022 has shown mostly AgV3 variant in human cases with 29/45 (64.4%) reports including 23 from bats, four from cats, and two from unknown species, followed by 8/45 (17.8%) of AgV2 variant (all from dogs), 4/45 from marmoset variant (all from *Callithrix jacchus*), 2/45 samples compatible with wild canid variant (both from *Cerdocyon thous*), and one/45 of AgV1 variant from a domestic dog. Only one sample of human rabies was not typified, related to bat aggression. In addition, surveillance conducted in the state of São Paulo confirmed the presence of rabies in 7/23,839 cats (0.031%) and 3/106,637 dogs (0.003%) between 2003 and 2013, with a 10:1 overall cat-to-dog positivity ratio. This 10-fold higher infection rate for cat rabies may be explained by cats’ hunting habits and predation. In addition, after 28 years of rabies-free status, a new cat rabies case was reported in the city of São Paulo in 2011. The rabid cat lived, along with other pets, in a household located near the largest downtown city park, whose owners presented animal hoarding behavior. Thus, animal hoarders and rescuers, public health agents, animal health professionals, and the general population with contact need to be aware of the risk of bat-borne rabies followed by spillover from cats to humans. In conclusion, cat rabies cases are becoming increasingly important in Brazil. This poses a One Health concern, given the overlapping of human, bat and cat populations within the same predisposed environment.

## 1. Introduction

Rabies, caused by the virus *Rabies lyssavirus*, causes severe and invariably fatal encephalitis in mammalian species (1). Among the most neglected tropical diseases (2), rabies has two major transmission routes implicated in tropical countries: an urban cycle with dogs and cats; and a sylvatic cycle, mainly through bats (3).

Rabies in domestic cats has been described far less often than dogs (4–7). Massive vaccination programs controlled dog rabies, but cats were less vaccinated, mostly due to difficulties in transporting and restraining cats, along with underestimation of feral cats (8, 9). As cats are considered dead-end hosts, human rabies by cats is rare (10). Nonetheless, bat rabies increased in urban areas, mostly due to anthropization (11). Overlapping habitats of cats and bats may lead to contact and rabies transmission (12). Rabies virus has been detected in cat salivary gland and brain tissues (13). Reports on predation episodes indicated spillover risk of rabies transmission from bats to cats (14, 15).

Cats may be a spillover-spreading route for rabies, with high likelihood of bat contact (4, 11). Recent cases of human rabies by cats were reported in Argentina (16) and Colombia (17), nearby South American countries. Brazilian cat population was estimated in 22.1 million, with 14.1 (19.3%) million households presenting at least one cat (18). Central and South America are the home of almost one third of the world’s bat species, overlapping in Brazil with the largest South American dog and cat population. Accordingly, the aim of the present review was to assess the data available on rabies in humans and companion animals in Brazil, including cases of human rabies transmitted by cats, and cases of rabies in cats.

## 2. Methods

The present study consisted of a review of animal and human rabies cases notified by all municipal health departments (rabies is a compulsorily notifiable disease in Brazil) between 1986 and 2022. This review was based on the data available from the epidemiological health surveillance system (DATASUS) of the Brazilian Ministry of Health. It summarizes historical cases of human and companion animal rabies in Brazil, including the cases of human rabies transmitted by cats, and cat rabies cases. Additionally, a cat rabies case notified in 2011 in the city of São Paulo, the city with the largest population in Latin America, is briefly described here. All the data used in this study were obtained from sources available in municipal and state health departments, as well from the Brazilian Ministry of Health, and have been anonymized.

## 3. Reports of human rabies due to feline transmission in Brazil

Between 2010 and 2022, a total of 45 fatal human cases of rabies were reported in Brazil, including 24 directly by bats, nine by dogs, four by cats, four by non-human primates, and two by foxes, along with two of unknown origin (19).

During the period from 1986 to 2000, 28 cases of human rabies transmitted by cats were recorded. Although variant typing was not a common practice in diagnostic laboratories during that time, most of the cases are likely to be associated with the AgV2 variant of canine origin since the majority of cases occurred in regions where this viral variant was endemic. According to Araújo (2002) (20), during the period from 1992 to 2000, there were 16 cases of human rabies transmitted by cats, with the majority occurring in the Northeast region (11 out of 16 cases).

A total of 28 human rabies cases transmitted by cats was recorded from 1986 to 2000 in Brazil, with 16 cases described from 1992 to 2000, mostly in the northeastern region with 11/16 (68.8%) reported cases (19). Although variant typing was not a common practice in diagnostic laboratories at the time, most cases were likely associated to AgV2 variant of domestic dog origin, as majority of cases occurred in regions where AgV2 variant viral variant was endemic. Out of the six human rabies cases in Brazil due to cats over the past two decades, four occurred in the northeastern and Northern regions. In addition, the first case of cat infection with variant 3 virus (AgV3), commonly found in vampire and frugivorous bats, was reported in southeastern Brazil in 2001; and, most recently, a human rabies case caused by a cat was reported in the Southern region in 2019 (Table 1). This form of transmission, known as a secondary cycle (bat → cat → humans) has been recorded in five human rabies cases in Brazil, and a summary of the information available on these cases, including municipality, year, virus variant, and form of transmission (exposure), has been gathered and presented (Table 1).

**Table 1:** Cases of human rabies associated with aggression by cats (*Felis catus*) between 2001 and 2022.

Year	Municipality—state	Viral variant	Age/sex	Exposure	Type of cat	Area
2001	Dracena—SP	Bat, AgV3	52 years/female	Bite (right hand)	Pet*	UNK
2004	Vitorino Freire—MA	UNK	15–19 years/female	UNK	UNK	UNK
2015	Jacaraú—PB	Bat, AgV3	1 year 8 min/male	Bite	Pet	Rural
2016	Boa Vista—RR	Bat, AgV3	14 years/male	Bite	Pet	UNK
2017	Recife—PE	Bat, AgV3	36 years/female	Bite (right breast)	Stray	Urban
2019	Gravatal—SC	Bat, AgV3	58 years/female	Bite	Pet	Rural

UNK, unknown; AgV3, *Desmodus rotundus*/*Artibeus lituratus*; ^*^With previous bat contact.

## 4. Reports of cat rabies in Brazil

**Figure 1:**
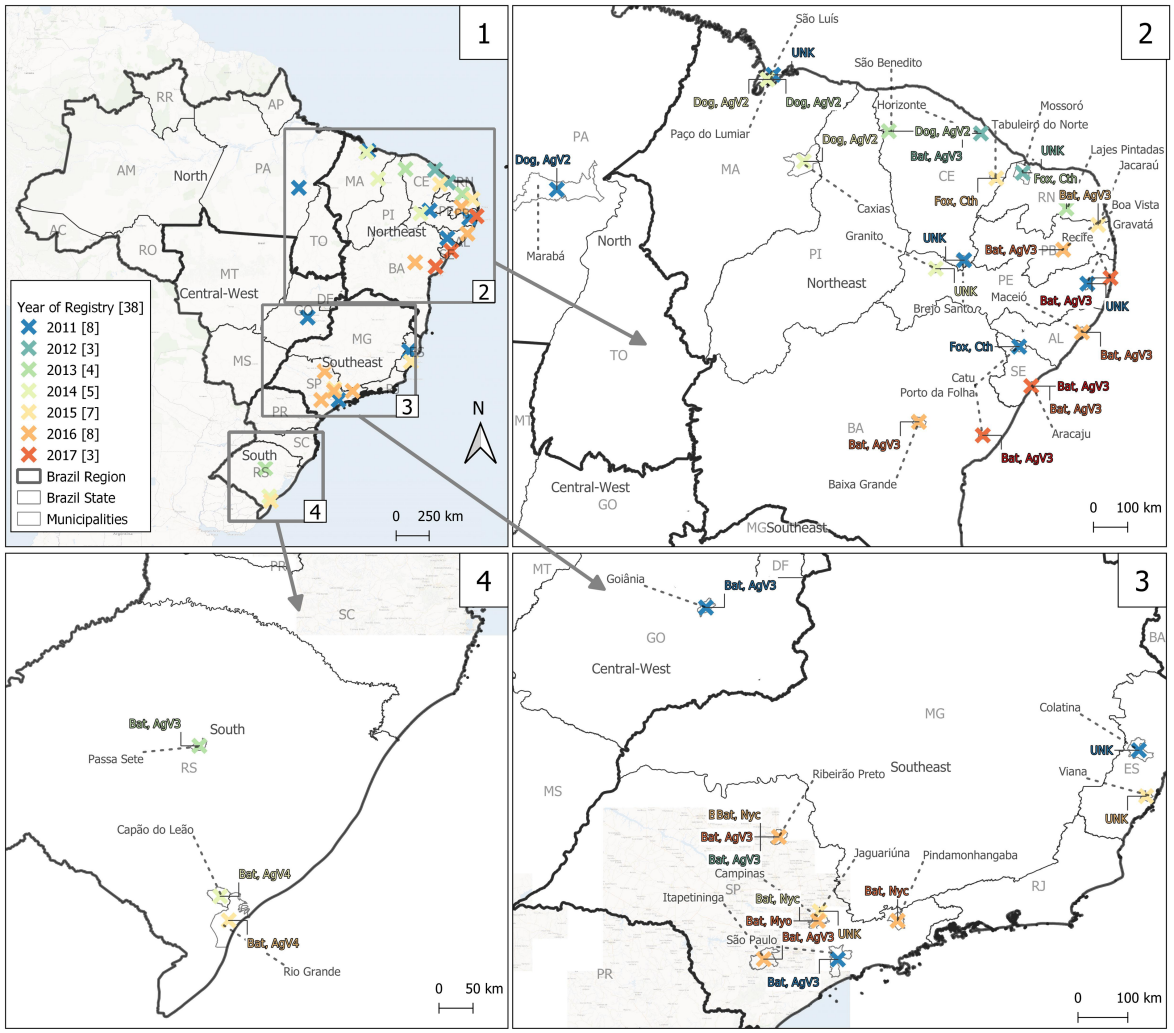
Positive rabies samples of domestic cat (*Felis catus*) and their associated variants in Brazil from 2011 to 2022.

Cat rabies cases between 2011 and 2022 were mostly caused by the variant 3 virus (AgV3), commonly found in bats and accounted for 36/72 cases (50.0%). Out of the 36 remaining cases, 25 were caused by other rabies variants and 11 were unknown, as presented (Figure 1; Supplementary Table 1). The number of these cases each year varied, as follows: eight (2011), three (2012), four (2013), five (2014), seven (2015), eight (2016), four (2017), two (2018), nine (2019), three (2020), 10 (2021), and nine (2022; Supplementary Table 1). Also, the geographic location varied, with AgV3 cases mostly often reported in southeastern region, with 16/36 (44.4%) cases, northeastern with 13/36 (36.1%), Central-Western with 4/36 (11.1%) and Southern region with 3/36 (8.3%; Supplementary Table 1).

Out of the 25 cases caused by other variants, 12 (nine since 2020) crab-eating fox variant (*Cerdocyon thous*) cases were from wild dogs and primarily found in northeastern region; five domestic dog-borne variant 2 (AgV2) reported between 2011 and 2014 in the northeastern and Northern regions; all four cat cases caused by variant 4 (AgV4, from insectivorous bat species *Tadarida braziliensis*) were found in Southern Brazil; all three cases caused by *Nyctinomops* spp. variant (another insectivorous bat) were in São Paulo state, southeastern; and finally a single isolated case of *Myotis* spp. variant (insectivorous bat species) in southeastern. The crab-eating fox (*Cerdocyon thous*) variant, which probably originated from wild dogs and is primarily found in the northeastern Brazil, was found in 12 infected cats during the period (nine cases since 2020). Lastly, the domestic dog-borne variant 2 (AgV2) rabies strain has not been reported in cats in Brazil for several years, such that the last five cat cases were between 2011 and 2014, all reported in the northeastern and Northern Brazil.

## 5. Reports of cat rabies in the state of São Paulo

Surveillance in São Paulo state showed that 7/23,839 cats (0.031%) and 3/106,637 dogs (0.003%) tested between 2003 and 2013 were confirmed rabies cases, with overall cat-to-dog ratio of almost 10:1 (0.031:0.003%) (21). This 10-fold higher likelihood may be related to cats’ hunting habits and predation, associated with overlapping of bat and feral cat populations in Brazil.

## 6. Report of cat rabies in the city of São Paulo after 28 rabies-free years

A new cat rabies case was reported in downtown São Paulo in 2011, after 28 years of rabies-free status, since 1983 (22) (Supplementary Table 1). A 10-year-old neutered female cat suddenly died with suspected poisoning. Cat had unconfirmed rabies vaccination in 2010, lived along with 23 other cats and five dogs with outdoor access, nearby the largest downtown wooded city park (Ibirapuera). The three household family members displayed hoarding behavior and were attended by city healthcare services. Five cats were for rabies observation, while others were monitored at household in cat cages. All cats were considered unhealthy, weak, and starving. City services decided to provide cat care and treatment at household to avoid stress and disease spreading; only the unhealthiest cats were taken. Owners reported cat contact with bats. At necropsy, cat stomach was empty, only containing cat hair by self-licking. Lack of granules and negative carbamate results in cat stomach was followed by brain tissue sent for rabies passive surveillance, with positive result later confirmed by the Pasteur Institute of São Paulo. Immediately after confirmation, massive anti-rabies vaccination campaign was applied to entire park area. The rabies sample was characterized at as variant 3 (AgV3), mostly found in *Desmodus rotundus* (hematophagous bat species) and *Artibeus* spp. (non-hematophagous bat species). The herein positive rabies cat from the park was the first report after a history of rabies-free for 28 years.

## 7. Discussion

This study presents historical cases of human rabies caused by infected cats and cases of cat rabies that have been reported in Brazil. Historical surveillance of rabies cases among companion animals in São Paulo state showed an overall 10-fold higher likelihood of rabies infection in cats than dogs. After 28 years of disease-free status, cat rabies was detected in downtown São Paulo, the largest city and urban area in Latin America (fourth largest worldwide), followed by a cat rabies case in Curitiba city after 29-year disease-free period (6) and recent cat rabies case in Santa Catarina state, Southern Brazil, after 38-year disease-free status since 1981 (23). Although cats may encounter vampire bats as well, no rabies case has been associated with hematophagous (vampire) bat variant 3 (AgV3). Monitored and eradicated from urban areas nationwide, hematophagous bats may hardly encounter cats during their lifetime (11). However, as hematophagous bats have been responsible for several livestock rabies cases in rural areas, domestic and feral cats living in overlapping areas of their occurrence may be infected but misdiagnosed.

The bat species *D. rotundus* and *Artibeus* spp. have both been identified as important rabies virus reservoirs in Brazil (24). While vampire bats, which feed on livestock and human blood, have typically been found in habitats in rural areas, insectivorous and frugivorous bats have been identified as a potential source of rabies transmission in urban areas (25). Stray and feral cats living in urban areas mostly lack anti-rabies vaccinations and basic veterinary assistance, and they may be particularly exposed to bat-borne rabies, due to overlapping habitats and cats’ hunting habits (26).

As several other isolated cases have been reported nationwide, the cat rabies herein acquired from bats in the city of São Paulo in 2011, even after 28 years of no rabies report, should not be considered a total surprise. Nonetheless, whether the case was due to reemergence of infection or lack of testing, should be further investigated. A finding of a rabid cat in the city of Curitiba, Capital of the State of Paraná and the ninth biggest Brazilian city, about 410 kilometers (255 miles) South of São Paulo, after 29 years of rabies-free status, was also reported by our research group in 2010 (6). The last two human rabies cases registered in the State of Paraná were caused by a dog in Curitiba in 1975 and by a hematophagous bat (*D. rotundus*) in 1987. The last case of dog rabies registered in Paraná occurred in 1981. In the latter case, although direct immunofluorescence was negative, there was a positive biological test and further rabies virus characterization revealed variant 4, from *Tadarida braziliensis* bats. The reappearance of cat rabies in major Brazilian cities has highlighted the sylvatic-aerial risk of infection and thus highlights the need for bat monitoring in historically rabies-free areas (6). Likewise, in the state of Rio Grande do Sul, in Southern Brazil, an urban cat rabies case was detected in 1997 after an 11-year disease-free period (27). As already mentioned, reemergence of infection or lack of testing should be further investigated and fully established.

Bat rabies surveillance and associated risk factors for rabies spillover without human cases have been evaluated in Curitiba. A total of 1,003 requests for bat removal were made between 2010 and 2015, through which 806 bats were collected alive and were identified as belonging to 13 genera in three families (11). Among these, 387/806 (48.0%) were considered unhealthy and were sent for euthanasia and rabies testing, from which 9/387 (2.32%) were found to be positive. The nine positive bat cases included two specimens of genus *Molossus*, two of genus P*romops*, three of genus *Nyctinomops*, one of genus *Myotis*, and one of genus *Sturnira*. In addition, a total of 4,769 random suspicious samples were sent for rabies diagnosis, including samples from dogs, cats, bats, and other animals, between 2007 and 2015. While all 2,676 samples of dog brain tissue tested negative, 1/1,136 (0.088%) cat brain tissue sample tested positive for rabies (11). Although these data showed that the prevalence of rabies was very low, it was noted that the overlapping of bat and cat habitats in Curitiba might provide potential spillover pathways to human infection.

Such occasional reemergence or reintroduction of cat (and dog) rabies in rabies-free areas may be caused by pet movements. This may even form a risk of for international reintroduction as well (27). In enzootic rabies areas, annual incidence potentially greater than 1.5% in dogs and 0.15% in cats has been predicted in high-burden areas of Africa and Asia, but much lower levels in Latin America. Unfortunately, wildlife can still potentially infect dogs and cats through spillover events, thereby increasing the risk of infection. Despite no registered human or pet rabies cases for almost 30 years in São Paulo and Curitiba, the rabies virus was continuing to circulate in both cities, with annual reports of positive bats (6). In 2022, 83/5,411 non-hematophagous bats (1.5%) were diagnosed as positive for rabies in São Paulo state.

The result shown in the present study of 34/766 human rabies cases (4.4%) from 1986 to 2022 directly related to cat transmission, reported as aggression by infected species and molecular typing of RABV strain, was 2.6 times higher in absolute case numbers and 1.5 times higher in percentage terms than in South Africa, over a similar 36-year period from 1983 to 2018. However, as no reports were made of bat transmission in unapparent or non-noticed bites or scratches, this has been a limitation in the present study. In South Africa, 13/458 human rabies cases (2.84%) were linked to domestic cat exposure (10). This comparison of results between these two tropical countries may indicate that cats play a more important role in human rabies in Brazil than in South Africa. The reason for this difference may be that hematophagous (vampire) bats, which are considered to be important reservoirs and transmitters for maintenance of sylvatic rabies in natural areas, have only been reported as inhabiting the Americas, with no occurrence in Africa. In addition, while the cat population in South Africa has been estimated as 2.4 million cats (proportionally, 4.0% of the country’s 59.4 million human inhabitants), the cat population in Brazil is more than 10 times higher, reaching around 27.1 million cats (12.6% of the country’s 214.3 million human inhabitants). As already mentioned, this high population and overlapping habitats may predispose toward bat hunting by cats, with a higher likelihood of bat-to-cat transmission in Brazil than in South Africa (12). Other rabies studies of inter-species dynamics in South America have shown that dog accounted for 40 human cases in Bolivia, nine in Brazil, six in Peru, two in Venezuela, and one in Chile, while cats caused single human infections in Brazil and Ecuador from 2009 to 2018 (3). In addition, as Argentina and Brazil have stopped dog and cat vaccination campaigns in recent years, rabies transmission from bats to pets and subsequently to humans has been a growing concern, making bat surveillance a crucial monitoring and control measure (3).

As already suggested for Southern Brazil, bats have been considered to be less vulnerable to forest fragmentation than other mammal species in these areas and may disperse and adapt to peri-urban or urban areas (28). Despite anthropization changes, increase of livestock populations in rural areas have been provided blood supply for vampire bats (29). Moreover, insectivorous bats hunting insects attracted by city lights may have favor inter-bat species transmission of rabies. As observed herein, bats may also transmit rabies virus to other mammal species such as dogs and cats (28). As presented in this study, massive vaccination campaigns may have switched the importance of active and passive rabies surveillance from pets to bats in Brazil.

Cats were also found to be 4.8-fold more likely to come into contact with bats than were dogs, in rabies testing done in Canada (30). Out of a total of 6,258 bats tested between 2014 and 2020, 41.5% had had encounters with cats (among which 91.1% were free-roaming and 8.9% were indoor cats) and 8.7% had had encounters with dogs (28). Although the results indicated that the highest probability was that rabies-positive bats had had encounters with dogs (20.2%), followed by no encounters (no animal exposure; 16.7%) and encounters with free-roaming cats (6.9%), cats with unspecified histories (6.0%) and non-free-roaming (indoor) cats (3.8%), the cumulative fivefold higher cat-bat interaction led to higher overall rabies exposure risk among humans from any free-roaming outdoor cats (28). Thus, for the rabid cat in the city of São Paulo described in this review, its access to the outdoors and presence of rabies variant 3 (AgV3), which is likely to have originated from bats, were highly expected.

The environment role in rabies has been a controversy matter regarding the One Health approach, given that rabies has been reportedly spread exclusively through direct transmission, mostly by direct body fluid contact during animal-to-animal interaction, without environmental involvement. However, changes caused by anthropization may have provided a better environment for rabies transmission, due to overlapping contact between humans, domestic animals, and wildlife. In a study conducted in Canada, humans were less likely to be exposed to rabid wild animals, but more likely to be exposed to rabid dogs and cats after these had been in contact with wildlife, most commonly rabid skunks (*Mephitidae* spp.) (30). On the other hand, massive animal vaccination programs worldwide have created an “environmental barrier” of immune protection (31, 32). The source of rabies transmission to humans has thus switched from vaccinated to unvaccinated animals, i.e., from dogs to bats and cats. Due to rabies control and low case reports, Southern states of Santa Catarina and Rio Grande do Sul have discontinued vaccination campaigns against rabies in dogs and cats, and Paraná state has performed Brazil-Paraguay border vaccination until 2015. Out of the 23/27 Brazilian states that performed vaccination campaigns against rabies in 2021, only 12 submitted performance data and overall vaccination was estimated in 60.4% coverage. Due to COVID-19 pandemics in 2020, São Paulo and Tocantins states, and other 219 municipalities of other states have not performed vaccination campaigns (33).

Lastly, the cat rabies case reviewed above, in a household with three humans who presented hoarding behavior, highlights that the close contact that individuals in this vulnerable population have with their animals gives rise to a potential risk of rabies exposure. Our research group has estimated an overall ratio of 6.45 hoarding cases per 100,000 inhabitants in Curitiba, distributed throughout the city. These hoarders kept around 724 dogs and 390 cats, mostly under poor sanitary conditions, and these animals had outdoor access (34). Given that the estimated population of the city of São Paulo city is 6.32 times bigger than that of Curitiba, extrapolation would indicate that around 715 hoarding cases might exist in São Paulo, in which 4,575 dogs and 2,465 cats might be at risk of rabies, along with their hoarding owners. Moreover, other pet-contact groups might be at risk of bat-borne spreading of rabies and cat spillover to humans, including people involved in animal protection and rescue, healthcare agents and veterinarians.

In conclusion, cases of human rabies acquired from cats and cat rabies acquired from bats, with reemergence of cases in rabies-free areas, serve as a warning about the growing importance of cat rabies, particularly in major urban Brazilian areas. This signals a matter of concern within the One Health approach, due to overlapping of human, bat and cat populations living within the same predisposed environment.

## Author contributions

EM, PB, and PM: conceptualization. JL, EM, LK, LB, PB, AB, and PM: original draft preparation, writing, reviewing, and editing. PM: supervision. All authors contributed to the article and approved the submitted version.
