# Supplementary material for: Cat rabies in Brazil: a growing One Health concern
Source: Front Public Health. 2023 Jul 19;11:1210203. doi: 10.3389/fpubh.2023.1210203 (PMC10394693; doi:10.3389/fpubh.2023.1210203)
Supplement: Supplementary file 1 [file Table_1.docx]

Supplementary Material

Cat rabies in Brazil: a growing One Health concern

Jonathan Santos de Lima^1^, Enio Mori^2^, Louise Bach Kmetiuk^3^, Leandro Meneguelli Biondo^4^, Paulo Eduardo Brandão^1^, Alexander Welker Biondo^3^, Paulo César Maiorka^1^*

*** Correspondence:** Paulo César Maiorka; maiorka@usp.br

# Supplementary table

Supplementary Table 1. Domestic cat (*Felis catus*) rabies in Brazil between 2011 and 2022.

| Year | Municipality - state | Viral variant |
| --- | --- | --- |
| 2011 | Brejo Santo - CE | UNK |
| 2011 | Colatina - ES | UNK |
| 2011 | Goiânia - GO | Bat, AgV3 |
| 2011 | Gravatá - PE | UNK |
| 2011 | Marabá - PA | Dog, AgV2 |
| 2011 | Paço do Lumiar - MA | UNK |
| 2011 | Porto da Folha - SE | Fox, Cth |
| 2011 | São Paulo - SP | Bat, AgV3 |
| 2012 | Horizonte - CE | Bat, AgV3 |
| 2012 | Mossoró - RN | UNK |
| 2012 | Ribeirão Preto - SP | Bat, AgV3 |
| 2013 | Lajes Pintadas - RN | Fox, Cth |
| 2013 | Passa Sete - RS | Bat, AgV3 |
| 2013 | São Benedito - CE | Dog, AgV2 |
| 2013 | São Luís - MA | Dog, AgV2 |
| 2014 | Campinas - SP | Bat, Nyc |
| 2014 | Capão do Leão - RS | Bat, AgV4 |
| 2014 | Caxias - MA | Dog, AgV2 |
| 2014 | Granito - PE | UNK |
| 2014 | São Luís - MA | Dog, AgV2 |
| 2015 | Jacaraú - PB | Bat, AgV3 |
| 2015 | Jaguariúna - SP | UNK |
| 2015 | Ribeirão Preto - SP | Bat, Nyc |
| 2015 | Ribeirão Preto-SP | Bat, AgV3 |
| 2015 | Rio Grande - RS | Bat, AgV4 |
| 2015 | Tabuleiro do Norte - CE | Fox, Cth |
| 2015 | Viana - MA | UNK |
| 2016 | Aracajú - SE | Bat, AgV3 |
| 2016 | Baixa Grande - BA | Bat, AgV3 |
| 2016 | Boa Vista - RR | Bat, AgV3 |
| 2016 | Campinas - SP | Bat, Myo |
| 2016 | Itapetininga - SP | Bat, AgV3 |
| 2016 | Maceió - AL | Bat, AgV3 |
| 2016 | Pindamonhangaba - SP | Bat, Nyc |
| 2016 | Ribeirão Preto - SP | Bat, AgV3 |
| 2017 | Aracajú - SE | Bat, AgV3 |
| 2017 | Catú - BA | Bat, AgV3 |
| 2017 | Recife - PE | Bat, AgV3 |
| 2017 | Tabuleiro do Norte - CE | Bat, AgV3 |
| 2018 | Piracicaba - SP | Bat, AgV3 |
| 2018 | São José do Rio Preto - SP | Bat, AgV3 |
| 2019 | Aimorés - MG | Bat, AgV3 |
| 2019 | Barbalha - CE | UNK |
| 2019 | Governador Valadares - MG | Bat, AgV3 |
| 2019 | Gravatal - SC | Bat, AgV3 |
| 2019 | Ipiranga - PR | Bat, AgV3 |
| 2019 | Itaú de Minas - MG | Bat, AgV3 |
| 2019 | Mococa - SP | Bat, AgV3 |
| 2019 | Ourinhos - SP | Bat, AgV3 |
| 2019 | Quixeré - CE | Bat, AgV3 |
| 2020 | Boa Viagem - CE | UNK, AgV3 |
| 2020 | Capela do Alto Alegre - BA | Fox, Cth |
| 2020 | Rolador - RS | Bat, AgV4 |
| 2021 | Aguiar - PB | Fox, Cth |
| 2021 | Araci - BA | Fox, Cth |
| 2021 | Belo Horizonte - MG | Bat, AgV3 |
| 2021 | Brejo Santo - CE | Fox, Cth |
| 2021 | Caicó - RN | Fox, Cth |
| 2021 | Fartura - SP | Bat, AgV3 |
| 2021 | Igarassu - PE | Bat, AgV3 |
| 2021 | Paulo Frontin - PR | UNK, AgV3 |
| 2021 | Petrolina PE | Bat, AgV3 |
| 2021 | Sobral - CE | Fox, Cth |
| 2022 | Americana - SP | Bat, AgV3 |
| 2022 | Araripina - PE | Fox, Cth |
| 2022 | Campo Grande - MS | Bat, AgV3 |
| 2022 | Goiânia - GO | Bat, AgV3 |
| 2022 | Goiânia - GO | Bat, AgV3 |
| 2022 | Indaiabira - MG | Bat, AgV3 |
| 2022 | Jijoca de Jericoacoara - CE | Fox, Cth |
| 2022 | Moreilândia - PE | Fox, Cth |
| 2022 | Pelotas - RS | Bat, AgV4 |

UNK: unknown; AgV3: *Desmodus rotundus*/ *Artibeus lituratus*; AgV4: *Tadarida brasiliensis*; Cth: *Cerdocyon thous;* Myo: *Myotis* sp.; Nyc: *Nyctinomops* sp.
